# CRITICAL PERSPECTIVES ON VOLUNTARISM IN AGING RURAL COMMUNITIES: VOLUNTEER LEADERSHIP BIOGRAPHIES

**DOI:** 10.1093/geroni/igz038.062

**Published:** 2019-11-08

**Authors:** Mark Skinner, Alun Joseph

**Affiliations:** 1 Trent University, Peterborough, Ontario, Canada; 2 University Of Guelph, Guelph, Ontario, Canada

## Abstract

Voluntarism has been portrayed as a productive and even transformative process whereby rural communities, households and older residents are able to meet the challenges of changing rural demographics. Yet, little attention has been paid to building a critical perspective on the complex and often-contested expectations placed on older rural volunteers. This paper focuses on the particular gap in understanding the contributions of older rural adults as a crucial resource in creating opportunities for aging in place and sustainable rural community development. Drawing on research into voluntarism in Canada’s aging resource communities, this paper presents qualitative findings from innovative ‘volunteer leadership biographies’ with older residents who were involved in key voluntary sector initiatives to improve community development. The findings show how older volunteer leadership is embedded in both place (residency) and time (life course), revealing new dimensions to the problem of understanding volunteer leadership in an era of rural population change.

